# 6. A case of complex juvenile idiopathic arthritis in childhood, adolescence and adulthood

**DOI:** 10.1093/rap/rky031.005

**Published:** 2018-09-20

**Authors:** Ruth Smith, Rachel Tattersall

**Affiliations:** Rheumatology, Sheffield Teaching Hospitals, Sheffield, UNITED KINGDOM

**Introduction:** Juvenile idiopathic arthritis (JIA) is an umbrella term for inflammatory arthritis occurring before the age of 16 years. JIA is associated with several extra-articular manifestations including uveitis, macrophage activation syndrome, inflammatory bowel disease and psoriasis. JIA affects 1:1000 children and young people (CYP) and in over 50% has a lifelong course. Adolescence is a developmental stage occurring between 10 and 19 years and therefore adolescents with rheumatic disease are seen in both paediatric and adult rheumatology services. CYP with JIA have to negotiate both the turbulence and challenges of adolescence but also transition from paediatric to adult services where healthcare professionals are rarely trained in skills relevant to adolescence (communication skills, psychosocial screening, promoting autonomy and resilience) and may not understand that risky behaviours including non-adherence with treatment are normal in this stage. Young adults with chronic disease have many challenges including balancing the demands of treatment with establishing themselves in work, social relationships and building their own families. We present a 23 year old woman (A) who developed JIA aged two. Over the course of her life she has developed the extra-articular manifestations of uveitis, inflammatory bowel disease, macrophage activation syndrome and psoriasis and has refractory arthritis. She has been treated with all NICE approved JIA treatments (for which there is no upper age limit) and, given the refractory nature of her disease, with other biologic therapies too in close collaboration with gastroenterology and dermatology colleagues. She has remained resilient throughout and has contributed to this case study.

**Case description:** A was diagnosed in paediatric rheumatology with: polyarticular juvenile idiopathic arthritis aged two: rheumatoid factor and Anti CCP antibody negative, ANA positive with a homogenous pattern and ENA negative, treated with methotrexate 25mg weekly orally. Anterior uveitis aged six. Crohn’s disease aged 15: infliximab 6mg/kg monthly but she had only two doses before Crohn’s severity required a right hemicolectomy aged 17. Patient A was seen in a combined adult/paediatric rheumatology transition clinic for two years prior to transfer to adult services. At the time of transition in 2012, her joints were in remission and she hadn’t had an episode of uveitis for two years, but her Crohn’s disease was unstable. Although our patient had undergone a smooth transition from rheumatology paediatric to adult services, she was slow to engage with the adult gastroenterologists and missed several appointments, primarily because she was anxious about now having to undergo colonoscopies without general anaesthetic. Rheumatology led care in close collaboration with gastroenterology. A was on 25mg oral methotrexate weekly, but not tolerating this well due to nausea. Whilst being assessed for adalimumab to treat both JIA and Crohn’s, and despite dose reduction and a change to subcutaneous administration, A discontinued methotrexate herself in September 2013. A then had to postpone starting adalimumab due to episodes of tonsillitis. During this time her joints flared and she was manged with oral and intra-articular steroids - she could only tolerate joint injections with entonox inhaled anaesthesia. The provision of entonox as routine in young adult clinic had been key in her transition planning. A commenced 40mg adalimumab fortnightly in January 2014. She missed a number of doses due to further episodes of tonsillitis, but, after a six month trial of adalimumab her JIA worsened and it was discontinued. After much negotiation, 15mg subcutaneous methotrexate was restarted alongside sulfasalazine 1g BD. Just after transfer aged 18, A presented with acute Epstein Barr virus infection which was complicated by macrophage activation syndrome (MAS). She was treated with two pulses of 1g IV methylprednisolone and a reducing course of oral prednisolone and the MAS settled. A’s joints remained active and a new biologic agent was discussed. Etanercept was not an option because of the history of uveitis and Crohn’s. Infliximab was not favoured because of the inconvenience of having to attend for infusions - a key consideration as A had experienced significant disruption to her education and was desperate to self-administer medicines. A’s gastroenterologists felt that certolizumab would be a reasonable choice in the context of inflammatory bowel disease and although A was reluctant to start another biologic, having attended for numerous intra-articular and intra-muscular injections corticosteroid injections throughout the year, resolved herself to trying again. She had a fantastic response and her joints settled. She stopped methotrexate and sulfasalazine against advice to continue them. In June 2016 A had a severe relapse in her Crohn’s which necessitated admission and high dose steroids possibly precipitated by stopping her DMARDs, and azathioprine was introduced as an alternative. A’s Crohn’s remained active and a CT scan showed widespread inflammation in the sigmoid colon, terminal ileum and descending colon. She required further hospital admissions, intravenous/oral/enema steroids and exclusive enteral nutrition. Certolizumab was replaced with infliximab 5mg/kg. A developed an allergic reaction with swelling around the eyes, itching and throat tightness with the second infusion. She had developed high anti-drug antibodies (Remsima antibodies greater than 614). There was again discussion between the rheumatology and gastroenterology teams regarding the next appropriate biologic agent. Tocilizumab and abatercept had no evidence for inflammatory bowel disease and vedolizumab none for inflammatory joint disease. Ustekinumab was the remaining option, but required an individual funding request at the time. Fortunately this was granted and she started it in January 2017, alongside azathioprine 125mg OD and Prednisolone 40mg OD. After a month, A was experiencing some improvement in Crohn’s symptoms and began to taper prednisolone. An MRI showed colitis in the caecum and an inflamed right colon. Colonoscopy showed severe perianal disease. Surgical options were discussed and a loop ileostomy was considered the best option, leaving the colon in situ to help improve symptoms and avoiding pelvic dissection that might impinge on fertility. The procedure was done in April 2017, and ustekinumab was recommenced post-operatively alongside azathioprine 100mg OD. The surgery gave instant relief of the perianal symptoms and in our patient’s own words turned my life around. By August, A’s joints were flaring. Ustekinumab was stopped and certolizumab restarted. By the end of the year, both bowels and joints were flaring and she required hospital admission again. A CT scan showed mild to moderate inflammation from the transverse colon to the rectum, with the sigmoid looking most inflamed. She underwent a subtotal colectomy in February 2018 and for the first time began to experience profound depression. Again there was inter-departmental discussion regarding which biologic to try next should A’s JIA flare. Vedolizumab with apremilast or stem cell transplantation were considered. Ustekinumab was a possibility, but had not been truly effective before. Certolizumab was settled on, and reintroduced post-surgery at A’s request given its safety in pregnancy, which A was now starting to consider. In May 2018, A developed a psoriasiform rash around her stoma site after applying dansac soft paste to the dressing. The rash developed over twenty four hours and spread to involve the trunk, arms and neck. This was thought to be due to the Koebner phenomenon. Alongside this new development our patient is still getting synovitis, but is well from a bowel point of view and is gaining weight. She is currently on certolizumab monotherapy, planning a holiday to Bali and contemplating pregnancy.

**Discussion:** A has severe refractory JIA, but remains resilient and optimistic. This case exemplifies the importance of adolescent-friendly care, good transition and multidisciplinary working as well as the extra-articular manifestations of JIA. A writes: "living with juvenile arthritis from two years old has been crazy. For me once I was at the age where I began to realise my body and joints didn't work the way everybody else's did it became extremely frustrating. At around 14/15 I began to really understand arthritis. At the same time I started to realise that I wouldn't be with the same doctors forever at the children's hospital, that for me was very scary because I'd only just got my head around my condition. However, I started seeing my soon-to-be consultant in transition appointments alongside my current consultant. This was a massive relief for me, having the support and familiarity of someone I knew well and trusted whilst meeting someone new. Being still young and having to move hospitals, whilst battling a condition that is so aggressive, plus having to deal with scary new treatments and being at the age where you understand what's going on around you is overwhelming. Looking back at how I once found everything so confusing to now feeling confident enough to ask questions about my condition, share my worries about treatment and the future, I feel is all down to the way my rheumatology team have made me feel. It is so important that you don't just see your patient as another patient that you take time to build a trusting relationship and care about not just their condition itself but their overall needs. I'm very privileged to have that."

**Key Learning Points**: For the majority of people with JIA this is a lifelong disease but adult rheumatology training is sparse regarding the different joint distribution as compared with rheumatoid arthritis and characteristics of JIA. JIA has several severe extra-articular manifestations and in particular MAS is not only seen in systemic forms of JIA but should be actively considered in all unwell, febrile patients with JIA at any point in their life. Transition is a key issue for patients like A - she was very vocal about what worked for her: namely transferring care to rheumatology first and gaining confidence in adult services that has enable her to act autonomously in and direct and engage with her subsequent care. Adolescence is a distinct developmental stage with many stage appropriate behaviours including non-adherence with therapy and appointments and anxiety over new situations and treatments which can manifest as patients being demanding or difficult. Young-people friendly services need to be flexible and adaptable to these stage-appropriate behaviours eg chase up non-attendance and give as many choices as possible in therapies. With the advent of biologics like vedolizumab that work well in inflammatory disease but are not effective for arthritis, multi-speciality communication is key . We have developed a virtual biologic clinic with gastroenterology colleagues from which A has benefitted.

**Disclosure: R. Smith:** None. **R. Tattersall:** None.

